# 1,4-Phenyl­enebis(methyl­ene) dicarbamate

**DOI:** 10.1107/S1600536812012718

**Published:** 2012-03-31

**Authors:** Zhi Li

**Affiliations:** a Department of Chemistry, School of Science, Beijing Jiaotong University, Beijing 100044, People’s Republic of China

## Abstract

The title compound, C_10_H_12_N_2_O_4_, is a phenyl dicarbamate with crystallographically imposed inversion symmetry. The dihedral angle between the carbamo­yloxy plane [i.e. the plane of the N—C(O)—O fragment; r.m.s. deviation = 0.002 (3) Å] and the plane of the aryl ring is 29.2 (1)°. In the crystal, two different centrosymmetric N—H⋯O hydrogen-bond inter­actions are observed; these are described as *R*_2_^2^(8) and *R* ^2^_4_(8) in graph-set notation. The rings form an alternating sequence, linking the mol­ecules into a sheet structure parallel to (011).

## Related literature

For self-assembled monolayers of alkyl carbamate and alkyl dicarbamate, see: Kim *et al.* (2003 ▶); Kim *et al.* (2005*a* ▶,*b* ▶). For the synthesis of the title compound, see: Takeuchi *et al.* (1971 ▶, 1974 ▶).
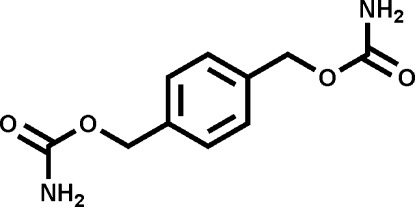


## Experimental

### 

#### Crystal data

C_10_H_12_N_2_O_4_
*M* *_r_* = 224.22
Triclinic, 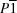

*a* = 4.9542 (14) Å
*b* = 6.4194 (18) Å
*c* = 8.418 (2) Å
α = 79.290 (4)°
β = 79.351 (4)°
γ = 88.640 (4)°
*V* = 258.50 (13) Å^3^
*Z* = 1
Mo *K*α radiation
μ = 0.11 mm^−1^
*T* = 294 K
0.30 × 0.28 × 0.22 mm

#### Data collection

Bruker SMART CCD area-detector diffractometer
Absorption correction: multi-scan (*SADABS*; Sheldrick, 1996 ▶) *T*_min_ = 0.962, *T*_max_ = 0.975
1310 measured reflections
902 independent reflections
764 reflections with *I* > 2σ(*I*)
*R*_int_ = 0.022

#### Refinement

*R*[*F* ^2^ > 2σ(*F* ^2^)] = 0.038
*wR*(*F* ^2^) = 0.100
*S* = 1.06
902 reflections
81 parameters
H atoms treated by a mixture of independent and constrained refinement
Δρ_max_ = 0.15 e Å^−3^
Δρ_min_ = −0.21 e Å^−3^

### 

Data collection: *SMART* (Bruker, 2007 ▶); cell refinement: *SAINT* (Bruker, 2007 ▶); data reduction: *SAINT*; program(s) used to solve structure: *SHELXS97* (Sheldrick, 2008 ▶); program(s) used to refine structure: *SHELXL97* (Sheldrick, 2008 ▶); molecular graphics: *Mercury* (Macrae *et al.*, 2008 ▶); software used to prepare material for publication: *Mercury* and *SHELXL97*.

## Supplementary Material

Crystal structure: contains datablock(s) global, I. DOI: 10.1107/S1600536812012718/nk2147sup1.cif

Structure factors: contains datablock(s) I. DOI: 10.1107/S1600536812012718/nk2147Isup3.hkl

Supplementary material file. DOI: 10.1107/S1600536812012718/nk2147Isup4.cdx

Supplementary material file. DOI: 10.1107/S1600536812012718/nk2147Isup4.cml

Additional supplementary materials:  crystallographic information; 3D view; checkCIF report

## Figures and Tables

**Table 1:** Hydrogen-bond geometry (Å, °)

*D*—H⋯*A*	*D*—H	H⋯*A*	*D*⋯*A*	*D*—H⋯*A*
N1—H1*A*⋯O1^i^	0.88 (2)	2.11 (2)	2.930 (2)	155.6 (17)
N1—H1*B*⋯O1^ii^	0.93 (2)	2.07 (2)	2.9888 (19)	169.8 (16)

Symmetry codes: (i) 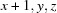; (ii) 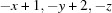.

**Fig. 1.**
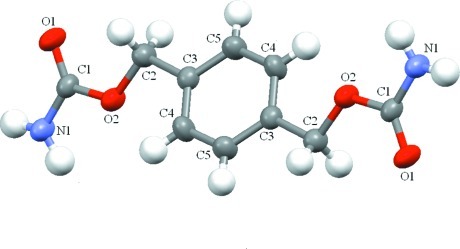
The structure of the title compound. Displacement ellipsoids are drawn at the 30% probability level.

**Fig. 2.**
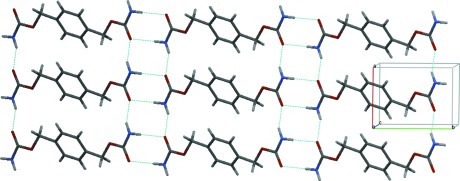
Packing diagram for (I). The dashed lines show N—H···O hydrogen bonds between neighboring molecules.

## supplementary crystallographic information

### Comment

Recently, self-assembled monolayers of alkyl carbamate and alkyl dicarbamate have been investigated and characterizd (Kim *et al.*, 2003, 2005*a*,b). For further study of the self-assembled activities of dicarbamates, herein, we report the synthesis and structure of a phenyl dicarbamate, 1,4-phenylenebis(methylene) dicarbamate (I) (Fig. 1). In (I), The dihedral angle between the carbamoyloxy plane [O1, C1, N1, O2 plane, mean deviation: 0.002 (3) Å] and the benzene plane is 29.2 (1)°. As shown in Fig 2, the O atom (O1 atom) of the carbonyl group acts as a double H-receptor. The two H atoms of the same amino group interact with the O atom (O1 atom) of the carbonyl group in the adjacent molecule to form two different intermolecular N—H···O hydrogen bonds (N1—H1A···O1 and N1—H1B···O1; Table 1). These are described as *R*_2_^2^(8) and *R*^2^_4_(8) in graph set notation. The rings are located in an alternating sequence to link the molecules into a two dimensional sheet structure.

### Experimental

The title compound was synthesized by transesterification of ethyl carbamate with 1,4-phenylenedimethanol (Takeuchi *et al.* 1971, 1974) as followed: A solution of 8.9 g (100 mmol) ethyl carbamate and 1.38 g (10 mmol) 1,4-phenylenedimethanol in 25 ml of toluene was heated to reflux in the presence of catalytic amount of zinc chloride for 10 h. After cooling to room temperature, the solvent was evaporated under vacuum. The residue was subjected to flash chromatography and the title compound was obtained as colorless crystal. (1.34 g, Yield: 60%; m.p. 484–486 K). Crystals suitable for single-crystal X-ray analysis were grown by slow evaporation of a DMF solution.

### Refinement

H atoms were placed in calculated positions [C—H = 0.93–0.97 Å] and allowed to ride on the parent atoms, with *U*_iso_ values constrained to be 1.2*U*_eq_ of the parent atom. The bond length of N1—H1A is 0.88 (2) Å and the bond length of N1—H1B is 0.93 (2) Å.

### Crystal data

**Table:**

C_10_H_12_N_2_O_4_	*Z* = 1
*M**_r_* = 224.22	*F*(000) = 118
Triclinic, *P*1	*D*_x_ = 1.440 Mg m^−^^3^
Hall symbol: -P 1	Melting point: 485 K
*a* = 4.9542 (14) Å	Mo *K*α radiation, λ = 0.71073 Å
*b* = 6.4194 (18) Å	Cell parameters from 819 reflections
*c* = 8.418 (2) Å	θ = 2.5–26.1°
α = 79.290 (4)°	µ = 0.11 mm^−^^1^
β = 79.351 (4)°	*T* = 294 K
γ = 88.640 (4)°	Needle, colourless
*V* = 258.50 (13) Å^3^	0.30 × 0.28 × 0.22 mm

### Data collection

**Table:**

Bruker SMART CCD area-detector diffractometer	902 independent reflections
Radiation source: fine-focus sealed tube	764 reflections with *I* > 2σ(*I*)
Graphite monochromator	*R*_int_ = 0.022
phi and ω scans	θ_max_ = 25.0°, θ_min_ = 2.5°
Absorption correction: multi-scan (*SADABS*; Sheldrick, 1996)	*h* = −4→5
*T*_min_ = 0.962, *T*_max_ = 0.975	*k* = −7→5
1310 measured reflections	*l* = −9→9

### Refinement

**Table:**

Refinement on *F*^2^	Primary atom site location: structure-invariant direct methods
Least-squares matrix: full	Secondary atom site location: difference Fourier map
*R*[*F*^2^ > 2σ(*F*^2^)] = 0.038	Hydrogen site location: inferred from neighbouring sites
*wR*(*F*^2^) = 0.100	H atoms treated by a mixture of independent and constrained refinement
*S* = 1.06	*w* = 1/[σ^2^(*F*_o_^2^) + (0.0539*P*)^2^ + 0.0526*P*] where *P* = (*F*_o_^2^ + 2*F*_c_^2^)/3
902 reflections	(Δ/σ)_max_ < 0.001
81 parameters	Δρ_max_ = 0.15 e Å^−^^3^
0 restraints	Δρ_min_ = −0.21 e Å^−^^3^

### Special details

**Table:**

Geometry. All e.s.d.'s (except the e.s.d. in the dihedral angle between two l.s. planes) are estimated using the full covariance matrix. The cell e.s.d.'s are taken into account individually in the estimation of e.s.d.'s in distances, angles and torsion angles; correlations between e.s.d.'s in cell parameters are only used when they are defined by crystal symmetry. An approximate (isotropic) treatment of cell e.s.d.'s is used for estimating e.s.d.'s involving l.s. planes.
Refinement. Refinement of *F*^2^ against ALL reflections. The weighted *R*-factor *wR* and goodness of fit *S* are based on *F*^2^, conventional *R*-factors *R* are based on *F*, with *F* set to zero for negative *F*^2^. The threshold expression of *F*^2^ > σ(*F*^2^) is used only for calculating *R*-factors(gt) *etc*. and is not relevant to the choice of reflections for refinement. *R*-factors based on *F*^2^ are statistically about twice as large as those based on *F*, and *R*- factors based on ALL data will be even larger.

### Fractional atomic coordinates and isotropic or equivalent isotropic displacement parameters (Å^2^)

**Table:**

	*x*	*y*	*z*	*U*_iso_*/*U*_eq_	
O1	0.2931 (2)	0.76540 (17)	0.12334 (15)	0.0498 (4)	
O2	0.5723 (2)	0.51407 (16)	0.22471 (13)	0.0427 (4)	
N1	0.7519 (3)	0.7787 (2)	0.02862 (18)	0.0451 (4)	
C1	0.5223 (3)	0.6939 (2)	0.12480 (18)	0.0361 (4)	
C2	0.3389 (3)	0.4099 (2)	0.3354 (2)	0.0419 (4)	
H2A	0.1959	0.3894	0.2749	0.050*	
H2B	0.2661	0.4963	0.4162	0.050*	
C3	0.4273 (3)	0.1990 (2)	0.41993 (17)	0.0348 (4)	
C4	0.6516 (3)	0.0921 (2)	0.35056 (19)	0.0432 (4)	
H4	0.7555	0.1532	0.2496	0.052*	
C5	0.2772 (3)	0.1045 (2)	0.57031 (19)	0.0417 (4)	
H5	0.1262	0.1741	0.6189	0.050*	
H1A	0.912 (4)	0.735 (3)	0.053 (2)	0.057 (5)*	
H1B	0.740 (4)	0.914 (3)	−0.031 (2)	0.054 (5)*	

### Atomic displacement parameters (Å^2^)

**Table:**

	*U*^11^	*U*^22^	*U*^33^	*U*^12^	*U*^13^	*U*^23^
O1	0.0332 (7)	0.0417 (7)	0.0668 (8)	0.0023 (5)	−0.0147 (5)	0.0152 (5)
O2	0.0355 (6)	0.0318 (6)	0.0531 (7)	0.0018 (4)	−0.0082 (5)	0.0120 (5)
N1	0.0345 (8)	0.0387 (8)	0.0538 (8)	0.0008 (6)	−0.0088 (6)	0.0135 (6)
C1	0.0359 (8)	0.0287 (8)	0.0421 (8)	0.0007 (6)	−0.0127 (6)	0.0029 (6)
C2	0.0373 (9)	0.0350 (9)	0.0468 (9)	0.0009 (6)	−0.0043 (7)	0.0060 (7)
C3	0.0364 (8)	0.0291 (8)	0.0373 (8)	−0.0008 (6)	−0.0086 (6)	−0.0001 (6)
C4	0.0470 (10)	0.0375 (9)	0.0368 (8)	0.0027 (7)	0.0021 (7)	0.0042 (6)
C5	0.0417 (9)	0.0348 (8)	0.0432 (9)	0.0071 (7)	−0.0005 (7)	−0.0013 (7)

### Geometric parameters (Å, º)

**Table:**

O1—C1	1.2163 (19)	C2—H2B	0.9700
O2—C1	1.3430 (17)	C3—C5	1.383 (2)
O2—C2	1.4348 (18)	C3—C4	1.386 (2)
N1—C1	1.331 (2)	C4—C5^i^	1.384 (2)
N1—H1A	0.88 (2)	C4—H4	0.9300
N1—H1B	0.93 (2)	C5—C4^i^	1.384 (2)
C2—C3	1.503 (2)	C5—H5	0.9300
C2—H2A	0.9700		
			
C1—O2—C2	116.37 (12)	C3—C2—H2B	109.9
C1—N1—H1A	119.3 (12)	H2A—C2—H2B	108.3
C1—N1—H1B	116.7 (11)	C5—C3—C4	118.33 (14)
H1A—N1—H1B	118.9 (16)	C5—C3—C2	119.38 (14)
O1—C1—N1	125.38 (14)	C4—C3—C2	122.27 (14)
O1—C1—O2	123.02 (14)	C5^i^—C4—C3	120.78 (15)
N1—C1—O2	111.59 (13)	C5^i^—C4—H4	119.6
O2—C2—C3	108.74 (12)	C3—C4—H4	119.6
O2—C2—H2A	109.9	C3—C5—C4^i^	120.89 (15)
C3—C2—H2A	109.9	C3—C5—H5	119.6
O2—C2—H2B	109.9	C4^i^—C5—H5	119.6
			
C2—O2—C1—O1	−1.5 (2)	C5—C3—C4—C5^i^	0.2 (3)
C2—O2—C1—N1	179.38 (13)	C2—C3—C4—C5^i^	−178.21 (15)
C1—O2—C2—C3	172.41 (12)	C4—C3—C5—C4^i^	−0.2 (3)
O2—C2—C3—C5	156.37 (14)	C2—C3—C5—C4^i^	178.25 (15)
O2—C2—C3—C4	−25.2 (2)		

Symmetry code: (i) −*x*+1, −*y*, −*z*+1.

### Hydrogen-bond geometry (Å, º)

**Table:**

*D*—H···*A*	*D*—H	H···*A*	*D*···*A*	*D*—H···*A*
N1—H1*A*···O1^ii^	0.88 (2)	2.11 (2)	2.930 (2)	155.6 (17)
N1—H1*B*···O1^iii^	0.93 (2)	2.07 (2)	2.9888 (19)	169.8 (16)

Symmetry codes: (ii) *x*+1, *y*, *z*; (iii) −*x*+1, −*y*+2, −*z*.

## References

[bb1] Bruker (2007). *SMART* and *SAINT* Bruker AXS Inc., Madison, Wisconsin, USA.

[bb2] Kim, K., Plass, K. E. & Matzger, A. J. (2003). *Langmuir*, **19**, 7149–7152.

[bb3] Kim, K., Plass, K. E. & Matzger, A. J. (2005*a*). *J. Am. Chem. Soc.* **127**, 4879–4887. 10.1021/ja043028+ 15796552

[bb4] Kim, K., Plass, K. E. & Matzger, A. J. (2005*b*). *Langmuir*, **21**, 647–655. 10.1021/la048299c 15641835

[bb5] Macrae, C. F., Bruno, I. J., Chisholm, J. A., Edgington, P. R., McCabe, P., Pidcock, E., Rodriguez-Monge, L., Taylor, R., van de Streek, J. & Wood, P. A. (2008). *J. Appl. Cryst.* **41**, 466–470.

[bb6] Sheldrick, G. M. (1996). *SADABS* University of Göttingen, Germany.

[bb7] Sheldrick, G. M. (2008). *Acta Cryst.* A**64**, 112–122. 10.1107/S0108767307043930 18156677

[bb8] Takeuchi, S. (1974). *Makromol. Chem.* **175**, 2241–2252.

[bb9] Takeuchi, S. & Ninagawa, E. (1971). *Bull. Chem. Soc. Jpn*, **44**, 3184–3185.

